# Time trends of major cancers incidence and mortality in Guangzhou, China 2004–2015: A Joinpoint and Age–Period–Cohort Analysis

**DOI:** 10.1002/cam4.3744

**Published:** 2021-03-16

**Authors:** Ao Luo, Hang Dong, Xiao Lin, Yu Liao, Binglun Liang, Long Chen, Guozhen Lin, Yuantao Hao

**Affiliations:** ^1^ Department of Medical Statistics and Epidemiology School of Public Health Sun Yat‐sen University Guangzhou China; ^2^ Department of Cause of Death and Cancer Surveillance Guangzhou Center for Disease Control and Prevention Guangzhou China; ^3^ Institute for Infectious Disease Control and Prevention Guangdong Provincial Center for Disease Control and Prevention Guangzhou China; ^4^ Government Affairs Service Center of Health Commission of Guangdong Province Guangzhou China; ^5^ Sun Yat‐sen Global Health Institute Sun Yat‐sen University Guangzhou China

**Keywords:** Age–Period–Cohort analysis, Guangzhou, incidence, mortality, time trend

## Abstract

**Background:**

Cancer is an important focus of public health worldwide. This study aims to provide a comprehensive overview of temporal trends in incidence and mortality of leading cancer in Guangzhou, China from 2004 to 2015.

**Methods:**

Data were collected from the population‐based registry in Guangzhou. Age‐standardized incidence rate (ASIR) and age‐standardized mortality rate (ASMR) were calculated and Joinpoint regression was used for evaluating the average annual percent changes (AAPC) among the entire study period and the estimated annual percent changes (EAPC) in time segments. The effects of age, period, and birth cohort were assessed by the age–period–cohort model.

**Results:**

The age‐standardized incidence and mortality by the world standard population decreased significantly among males with AAPC of −1.7% (95% CI: −3.0%, 0.2%) and −2.7% (95% CI: −4.3%, −1.1%) for all malignancies during 2004–2015, while among females, the age‐standardized incidence had a non‐significant reduction with AAPC of −1.3% (95% CI: −2.8%, 0.2%) and the age‐standardized mortality demonstrated a remarkable decline (AAPC −2.0%, 95% CI: −3.6%, −0.3%). For males, the most commonly diagnosed cancers were trachea, bronchus, and lung (TBL), liver, colorectal, nasopharyngeal, stomach, and prostate cancer. For females, breast, TBL, colorectal, liver stomach, and thyroid cancer ranked the top. Unfavorable trends were observed in ASIR of colorectal, thyroid, and prostate cancer. APC models yielded different ages, periods, and birth cohort effect patterns by cancer sites.

**Conclusions:**

Cancer burden remained a public health challenge in Guangzhou as the aging population and lifestyles changes, despite declines in incidence and mortality rates in some cancers. Surveillance of cancer trends contributed to valuable insights into cancer prevention and control.

## Graphical abstract

We aimed to evaluate the temporal trends of incidence and mortality rates in leading cancers by sex over the decade in Guangzhou, with an emphasis to analyze the detached effect of age, period of diagnosis, and birth cohort. And the study provided insight into the major cancer types with a comprehensive perspective in cancer variation trends and highlight the priorities that deserve attention for targeted intervention.

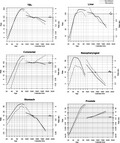

## 1 INTRODUCTION

Cancer, a significant public health concern worldwide, accounted for 24.5 million incident cases and 9.6 million deaths worldwide in 2017,^1^ and it has the second‐highest mortality rate following cardiovascular diseases.^2^ Incidence and mortality rates of cancer escalated in most regions of the world in the past decade.^1^ Over the past 40 years, China had a rapidly elevating cancer burden,^3^ and it is responsible for one‐fifth of global new cancer cases and more than a quarter of global cancer deaths.^4^ Patterns of leading cancers substantially altered across geographic regions, between countries, and within the country depending on the different degrees of socio‐economic and lifestyle factors.^4^ Lung, breast, and prostate cancer are the most common cancers in the United States and other developed western countries,^5^, ^6^ while lung, stomach, colorectal, liver, and female breast cancer represent the leading types in China, faced with a double burden of infection‐related cancers co‐existing with increasing rates of various lifestyle‐related cancers.^3^

Guangzhou is regarded as China's southern gateway to the world. As the well representative of frontier cities from 'Reform and Opening‐up' in China, Guangzhou has become one of the most highly‐developed metropolis globally, with a diverse culture and a prosperous economy. Being the second leading cause of death in Guangzhou, cancer represents a major challenge to public health in Guangzhou, as it was the second leading cause of death among inhabitants.^7^ The occurrence of cancer is attributed to multi‐factors, such as infectious agents, environmental characteristics, the local dietary habits, different lifestyles, and other racial, and familial aggregations.^8^, ^9^ Some regions are more prone to some specific cancer types, for example, the nasopharyngeal carcinoma in Guangdong Province.^10^ The rapid development of the economy and environment, changing fertility patterns, and increasing life expectancy are leading to cancer transition for countries at all levels of human development.^11^ Therefore, a better understanding of the epidemiological trends is critical to provide a basis for research on cancer pathogenesis and strategizing cancer control and prevention.^12^ Joinpoint regression analysis was widely utilized as a valuable tool in investigating trends of incidence and mortality rate over time, and the age–period–cohort model had the advantage of separating the temporal variations into three dimensions to investigate the independent effect of age, period, and birth cohort.^13^, ^14^, ^15^ Age effects are defined as variations associated with the individual age difference. Affecting all age groups simultaneously, period effects refer to influences of different observation periods, including a series of complex historical events, such as world wars, famine, and public health interventions. Cohort effects reflect the potential early life risk factors over generations and are evident in many cancer sites. Birth cohorts that experience different historical and social circumstances at different stages of their life course have various exposures to socio‐economic, behavioral, and environmental risk factors, and the essential role of early life exposures in predisposing the susceptibility to disease and mortality later in adulthood has been well elucidated.^16^

In this study, we aimed to evaluate the temporal trends of incidence and mortality rates of leading cancers by sex over the decade in Guangzhou, with an emphasis on analyzing the detached effect of age, period of diagnosis, and birth cohort. Meanwhile, the study is also designed to elaborate in‐depth insight into the most common malignancies with a comprehensive perspective in cancer variation trends and to highlight the priorities that deserve attention for targeted intervention.

## 2 **MATERIALS AND METHOD**S

### 2.1 Data sources

Guangzhou, the central prefecture of the Pearl River Delta that covers around 7500 square kilometers, consists of 11 districts with has a total permanent residential population of 13.5 million approximately in 2015. Cancer data of incident cases and deaths were derived from the Guangzhou cancer registry, coded in accordance with the 10th Revision of the International Classification of Diseases. Records on newly diagnosed cancer patients, including name, sex, date of birth, and the cancer type of diagnosis are routinely reported to the registry by medical facilities over the city. The crude data were coded and validated for eligibility by several comprehensive cross‐checking programs before registration, performing data cleansing and enhancement intra‐community, inter‐community, and cross‐district. The quality and accuracy of the registration data in Guangzhou were widely acknowledged. With the fulfillment of strict selective criteria, its data have been accepted and published by the China Cancer Registry Annual Report and the volumes of Cancer Incidence in Five Continents.^17^, ^18^ The proportion of submissions with morphological verification (MV%) reached 75.8%–76.1%, the proportion of submissions with a sole death certificate spanned 0.5–0.6, and the mortality‐to‐incidence ratio (M/I) totaled 53%–57%,^7^, ^19^ meeting all quality specification.

Briefly, new cases of cancer diagnosed in this study were collected from the whole city during 2004–2015, and cancer deaths for 2004–2015, covering six administrative districts in Guangzhou, including Yuexiu, Liwan, Haizhu, Huangpu, Luogang, and Baiyun Districts. The de‐identification process was performed to enhance privacy and ensure data sensitization. We defined the mid‐year population data as the total population of the year to evaluate the rates of incidence and mortality. Annual population data in the analysis during the corresponding period were obtained from the Guangzhou Statistics Bureau.^20^ Our study complies with the Guidelines for Accurate and Transparent Health Estimates Reporting (GATHER) statement. Data retrieved from the Annual Report of Guangzhou Cancer Registry are publicly available on the official website of Guangzhou Municipal Center for Disease Control and Prevention.^21^ The requirement for ethical board approval was waived because of the retrospective nature of this study and the anonymity of individual information.

### 2.2 Statistical analysis

First, we calculated the cancer incidence and mortality rates stratified by sex, cancer site, age group (progressive 5‐year age groups) during 2004–2015. Age‐standardized incidence rate (ASIR) and age‐standardized mortality rate (ASMR) were computed by applying the direct standardization method with the world standard population proposed by Segi and later modified by Doll et al.^22^, ^23^ The major cancer types and the leading causes of cancer deaths were recognized based on the data, with the trachea, bronchus, and lung (TBL), liver, colorectal, and stomach cancer among both men and women, nasopharyngeal and prostate cancer for men, while breast and thyroid cancer for women. The cancer rates for males and females were further analyzed separately to evaluate the sex differences.

To examine temporal trends of leading cancer incidence and mortality rates, Joinpoint regression analysis was performed to identify the year with remarkable pattern changes. ASIR and ASMR were transformed by logarithmic transformations in the model and the standard errors were calculated based on binomial approximation. The average annual percent change (AAPC), the estimated annual percent change (EAPC), and the corresponding 95% confidence interval (CI) for each trend segment were evaluated by the Joinpoint regression model.^24^ The significant tests were performed using the Monte Carlo permutation method and the Bayesian Information Criterion (BIC) was recruited to determine the optimized joinpoints.^25^ The analysis was conducted using the Joinpoint regression program software, version 4.8.0.1 (National Cancer Institute, USA).^26^

Age–period–cohort (APC) regression model was conducted to investigate the different effects of age, period, and cohort on the incidence and mortality trends for common cancers.^27^, ^28^ In the APC model, data were aggregated cases and population for 14 five‐year age groups (20–24 years to 85+ years) and 12 one‐year periods (from 2004 consecutively to 2015), in which we excluded the groups aged under 20 years old because of few cases in cancers among young age groups. The linear dependence among the factors of age, period, and cohort makes the model unidentifiable. To overcome dependence, Holford suggested a parameterization method by setting constraints on the parameters.^15^, ^29^ We constrained cohort effects to be 0 on average with 0 slope, and the inclusion of the drift with the period effect makes the age effect interpretable as the age‐specific rates in the reference period adjusted by the cohort effect. Based on a log‐linear model with Poisson distribution, the classic APC model was expressed as follows:

$$\log\left[\lambda \left(a,p\right)\right]=f\left(a\right)+g\left(p\right)+h\left(c\right)$$

where *λ* (*a*, *p*) refers to the incidence or mortality at the age *a* in period *p* for persons in birth cohort *c* = *p*‐*a*, and *a*, *p* and *c* represent the mean age, period, and cohort for the observational units. The model was fitted using natural cubic splines and maximum likelihood estimation, and the goodness of model fit was tested by comparing the residual deviance. The relevant sub‐models were arranged into a sequence to provide relevant comparisons of the linear, non‐linear cohort, and period effects. Significant curvatures in cohort and period effects were detected by comparing the differences in deviances with the degree of freedom using the Chi‐square test.^28^ A two‐sided *p* value of less than 0.05 was considered statistically significant. The APC analysis was performed using the EPI package in R software (version 3.6.2).^30^

## 3 RESULTS

### 3.1 Descriptive analysis of common cancers by sex

Descriptive data with base characteristics for this study are summarized in Table 1. A total of 142,526 men and 119,674 women were diagnosed with invasive cancers during 2004–2015 in the entire city of Guangzhou, resulting in the deaths of 52,847 men and 31,255 women within the six cancer registries. In 2015, TBL was found to be the most common cause of both cancer incidence (19.6% of all cases) and cancer death (23.5% of the total cancer deaths) for both sexes combined. Accounting for 55.5% of total cancer diagnoses, the top three cancers with the highest incidence and mortality among males were TBL (3258 new cases and 1658 deaths), liver (2097 new cases and 873 deaths), and colorectal cancer (2042 new cases and 618 deaths). For females, 47.4% in our study were diagnosed with the three most commonly diagnosed cancers, which were breast, TBL, and colorectal cancer, recording 2429, 1760, and 1550 diagnoses, respectively. For cancer mortality among females, TBL, colorectal, and breast cancer were the most common causes, resulted in 778, 469, and 334 deaths, respectively. The incidence of prostate and thyroid cancer ranked fourth for incidence among men and women, respectively.

**TABLE 1:** Incidence and mortality rates (per 100,000) of the most common cancer type by sex in Guangzhou, 2004–2015.

Sex	Cancer site	Incidence			Morality		
		Case	CDR	ASR	Case	CDR	ASR
Male	All site	142,526	295.46	226.77	52,847	217.30	149.31
	TBL	32,433	67.23	50.21	17,213	70.78	47.56
	Liver	21,540	44.65	34.27	10,008	41.15	29.07
	Colorectal	18,603	38.56	28.81	5286	21.74	14.32
	Nasopharyngeal	9391	19.47	14.99	2606	10.72	7.75
	Stomach	7284	15.10	11.38	2783	11.44	7.66
	Prostate	6408	13.28	9.25	1531	6.30	3.78
Female	All site	119,674	255.83	182.61	31,255	132.19	79.69
	Breast	23,957	51.21	37.32	3045	12.88	8.44
	TBL	16,278	34.80	22.77	7992	33.80	19.19
	Colorectal	14,874	31.80	21.01	4236	17.92	9.82
	Liver	5331	11.40	7.67	2889	12.22	7.29
	Stomach	4158	8.89	6.02	1768	7.48	4.40
	Thyroid	7142	15.27	11.63	183	0.77	0.44

Abbreviations: ASR, age‐standardized rate by Segi's World Standard Population^22^, ^23^; CDR, crude death rates (per 100,000).

### 3.2 Joinpoint regression analysis in cancer incidence and mortality rates

**FIGURE 1:**
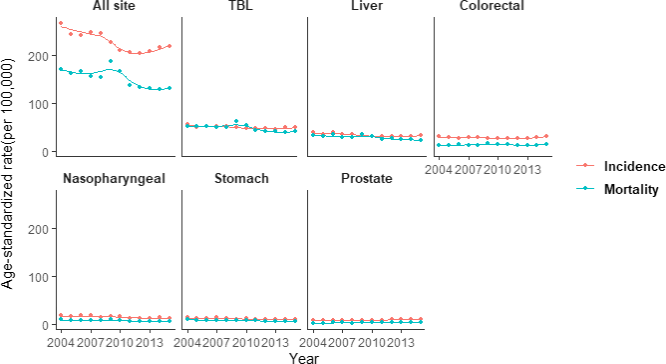
Age‐standardized incidence and mortality rates of six major cancers for males in Guangzhou, 2004–2015. TBL, trachea, bronchus, and lung.

**FIGURE 2:**
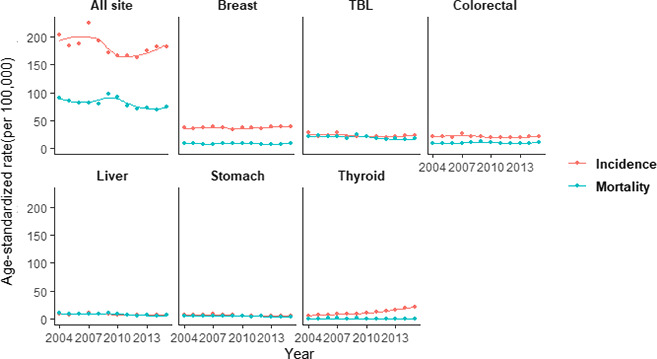
Age‐standardized incidence and mortality rates of six major cancers for females in Guangzhou, 2004–2015. TBL, trachea, bronchus, and lung.

The lifetime likelihood of developing cancer and the types of cancer at risk are different by sex. The scatter plots with LOWESS regression curves are presented in Figures 1 and 2, demonstrating the trends of ASIR and ASMR for common cancers by sex from 2004 to 2015. Joinpoint regression analysis indicated that both ASIR and ASMR decrease significantly among TBL, liver, nasopharyngeal, and stomach cancer on the overall trends during the decade, without detectable joinpoint in males. Meanwhile, the ASIR and ASMR of prostate cancer increased over the whole period, with the AAPC of 3.1% and 3.3%, respectively, showing significant changes. For colorectal cancer, the ASIR continuously decreased until 2012, followed by an unfavorable upward trend during 2012–2015 (EAPC 5.5%), which was statistically insignificant, while the ASMR remained relatively stable without significant change over the decade (AAPC 0.3%).

For ASIR among females, breast cancer remained at the highest level with an AAPC of 1.3% during 2004–2015, and similarly, colorectal, TBL, and liver cancer had non‐significant changes with AAPC of −0.9%, −1.5%, −2.3%, respectively, while a significant decline was observed in stomach cancer (AAPC −4.3%). However, there was a remarkable increasing trend with the AAPC of 13.3% of ASIR in thyroid cancer over the entire period. Apart from colorectal cancer, trends of ASMR were similarly illustrating continuously downward trends in the liver, stomach, TBL, thyroid, and breast cancer with AAPC of −4.3%, −3.3%, −3.0%, −1.7%, and −0.4%, respectively, while the latter two were not statistically significant. ASMR of colorectal cancer remained at a relatively high rate over the whole period (AAPC 0.7%), as described in Tables 2 and 3.

**TABLE 2:** Joinpoint regression analysis of trends in age‐standardized incidence and mortality rates of major cancers among males in Guangzhou, 2004–2015.

Cancer	Rate	AAPC (%, 95% CI)	Trend1		Trend2	
			Period	EAPC (%, 95% CI)	Period	EAPC (%, 95% CI)
All site	Incidence	−1.7^a^ (−3.0, 0.2)	2004–2012	−3.2^a^ (−4.4, −2.0)	2012–2015	2.6 (−2.8, 8.2)
	Mortality	−2.7^a^ (−4.3, −1.1)	2004–2015	−2.7^a^ (−4.3, −1.1)		
TBL	Incidence	−1.0 (−1.7, −0.3)	2004–2015	−1.0^a^ (−1.7, −0.3)		
	Mortality	−2.7^a^ (−4.6, −0.8)	2004–2015	−2.7^a^ (−4.6, −0.8)		
Liver	Incidence	−1.8^a^ (−2.8, −0.9)	2004–2015	−1.8^a^ (−2.8, −0.9)		
	Mortality	−3.3^a^ (−4.8, −1.7)	2004–2015	−3.3^a^ (−4.8, −1.7)		
Colorectal	Incidence	0.4 (−1.5, 2.3)	2004–2012	−1.5 (−3.2, 0.2)	2012–2015	5.5 (−1.5, 13.0)
	Mortality	0.3 (−1.7, 2.3)	2004–2015	0.3 (−1.7, 2.3)		
Nasopharyngeal	Incidence	−3.6^a^ (−5.1, −2.2)	2004–2015	−3.6^a^ (−5.1, −2.2)		
	Mortality	−4.0^a^ (−6.0, −2.0)	2004–2015	−4.0^a^ (−6.0, −2.0)		
Stomach	Incidence	−3.1^a^ (−4.3, −1.9)	2004–2015	−3.1^a^ (−4.3, −1.9)		
	Mortality	−4.3^a^ (−5.7, −2.8)	2004–2015	−4.3^a^ (−5.7, −2.8)		
Prostate	Incidence	3.1^a^ (2.1, 4.0)	2004–2015	3.1^a^ (2.1, 4.0)		
	Mortality	3.3^a^ (0.4, 6.3)	2004–2015	3.3^a^ (0.4, 6.3)		

Abbreviations: AAPC, the average annual percent change; EAPC, the estimated annual percent change.

^a^ AAPC or EAPC significantly different from 0 (two‐sided *p* < 0.05).

**TABLE 3:** Joinpoint regression analysis of trends in age‐standardized incidence and mortality rates of major cancers among females in Guangzhou, 2004–2015.

Cancer	Rate	AAPC (%, 95% CI)	Trend1	
			Period	EAPC (%, 95% CI)
All site	Incidence	−1.3^a^ (−2.8, 0.2)	2004–2015	−1.3 (−2.8, 0.2)
	Mortality	−2.0^a^ (−3.6, −0.3)	2004–2015	−2.0^a^ (−3.6, −0.3)
Breast	Incidence	−0.7 (−0.1, 1.4)	2004–2015	0.7 (−0.1, 1.4)
	Mortality	−0.4 (−2.1, 1.4)	2004–2015	−0.4 (2.1, 1.4)
TBL	Incidence	−1.5 (−3.5, 0.4)	2004–2015	−1.5 (−3.5, 0.4)
	Mortality	−3.0^a^ (−5.2, −0.8)	2004–2015	−3.0^a^ (−5.2, −0.8)
Colorectal	Incidence	−0.9 (−2.5, 0.9)	2004–2015	−0.9 (−2.5, 0.9)
	Mortality	0.7 (−1.4, 2.8)	2004–2015	0.7 (−1.4, 2.8)
Liver	Incidence	−2.3 (−4.6, 0.0)	2004–2015	−2.3 (−4.6, 0.0)
	Mortality	−4.3^a^ (−6.6, −2.0)	2004–2015	−4.3^a^ (−6.6, 2.0)
Stomach	Incidence	−4.3^a^ (−6.8, −1.6)	2004–2015	−4.3^a^ (−6.8, 1.6)
	Mortality	−3.3^a^ (−5.0, −1.6)	2004–2015	−3.3^a^ (−5.0, −1.6)
Thyroid	Incidence	13.3^a^ (11.4, 15.1)	2004–2015	13.3^a^ (11.4, 15.1)
	Mortality	−1.7 (−6.0, 2.7)	2004–2015	−1.7 (−6.0, 2.7)

Abbreviations: AAPC, the average annual percent change; EAPC, the estimated annual percent change.

^a^ AAPC or EAPC significantly different from 0 (two‐sided *p* < 0.05).

### 3.3 Age–period–cohort analysis

APC Poisson regression models were applied to evaluate the effects of age, cohort, and period on cancer incidence and mortality by sex. The full model (age–period–cohort model) yielded the best fit in the incidence and mortality rates of major cancers for both men and women with an exception. Age‐drift fits better for the incidence of prostate cancer in men, notwithstanding. The effects of thyroid cancer mortality rates were not detected in the model (See Appendix S1).

**FIGURE 3:**
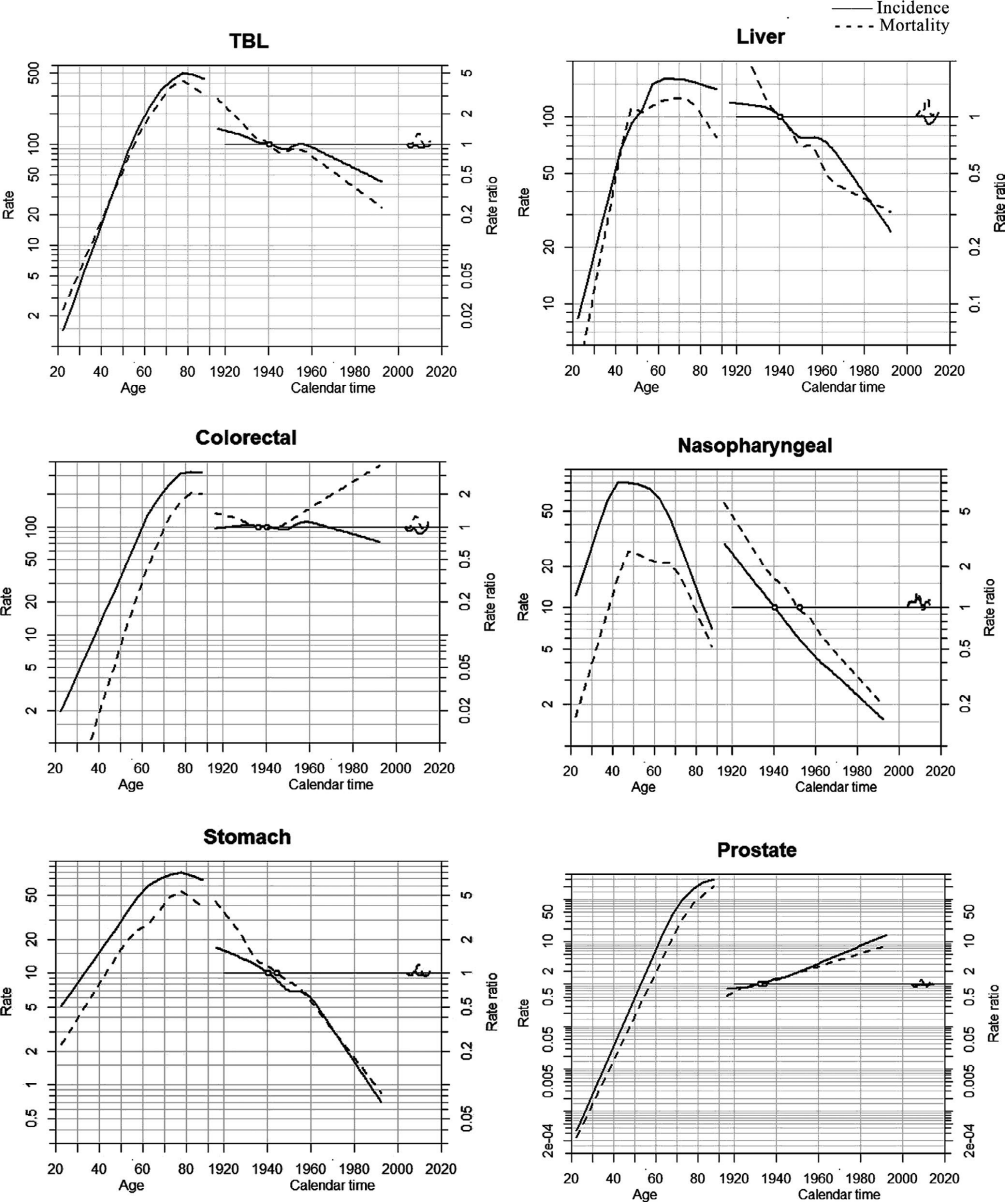
Age‐period‐cohort model of the incidence and mortality rates in major cancers among males in Guangzhou, 2004–2015. Notes: TBL, trachea, bronchus, and lung. Each graph includes three curves, with the solid line representing the incidence rate and the dotted line for mortality rate. From left to right, they are trends in the rates by age for the reference cohort (age effect), incidence risk by birth cohort (cohort effect), and incidence risk by calendar year (period effect). The graph has a horizontal axis divided into two parts: one for age (years old) and one for cohort‐period (calendar years). The left vertical axis represents incidence rates for the age effect, and the right vertical axis represents the relative risk for the cohort and period effect. The drift is added to the non‐linear birth cohort effects, and the right plot presents the period effect as residual ratio rates.

**FIGURE 4:**
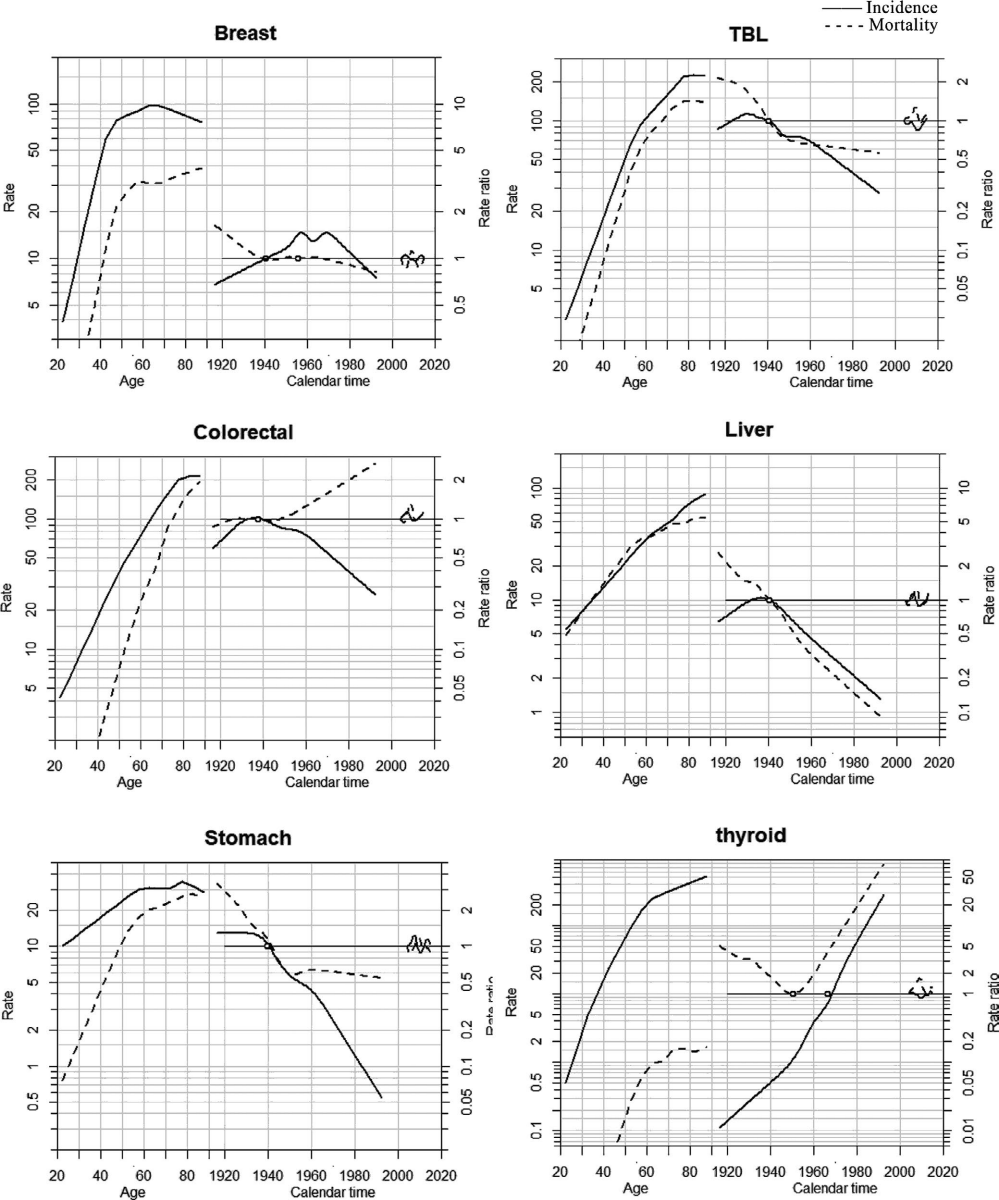
Age‐period‐cohort model of the incidence and mortality rates in major cancers among females in Guangzhou, 2004–2015. Notes: TBL, trachea, bronchus, and lung. Each graph includes three curves, with the solid line representing the incidence rate and the dotted line for mortality rate. From left to right, they are trends in the rates by age for the reference cohort (age effect), incidence risk by birth cohort (cohort effect), and incidence risk by calendar year (period effect). The graph has a horizontal axis divided into two parts: one for age (years old) and one for cohort‐period (calendar years). The left vertical axis represents incidence rates for the age effect, and the right vertical axis represents the relative risk for the cohort and period effect. The drift is added to the non‐linear birth cohort effects, and the right plot presents the period effect as residual ratio rates.

Figures 3 and 4 revealed the effects of age, period, and birth cohort by cancer sites according to sex. Both incidence and mortality consistently increased with advancing age in both sexes after 20 years old. For nasopharyngeal cancer among males, the highest age‐specific incidence and mortality rates were reported at aged 50 approximately. Meanwhile, peak incidence and mortality rates for females reached at the age of 60–69 year old for breast cancer and 70–79 year old for TBL, liver, colorectal, and gastric cancer. The incidence of prostate cancer remained relatively low until 50 year old for men; however, it escalated exponentially with age in the population over 50. The age‐specific mortality of prostate cancer indicated a similar propensity, as mentioned above. Regarding the cohort effects, cancer types that were common among males and females had similar trends, including TBL, liver, and stomach cancer. Incidence and mortality rates of these malignancies decreased in both sexes over the whole period and a falling risk of developing those cancers was observed in younger generations born no early than the 1940s in Guangzhou. However, the incidence of liver cancer among women increased slightly in the 1920–1940 cohort before declining. The incidence rate of colorectal cancer decreased continuously among men born after 1960 and among women born after 1940, and in contrast, the mortality reversed to elevate in successive birth cohorts from 1940. The incidence rate of breast cancer in women was accelerated in birth cohorts born before 1960 and then declined, while there was a rapid reduction of mortality rate in the cohorts born before 1940 and followed by a relatively gradual decline for succeeding birth cohorts. For females, the growing risk of developing thyroid cancer was discernable in birth cohorts consecutively.

## 4 DISCUSSION

Our study provides an up‐to‐date overview of temporal trends in incidence and mortality rates of the most common cancers by the population‐based cancer registration data in recent decades in Guangzhou. Overall, the age‐standardized incidence rate among males for all cancer sites combined decreased remarkably during 2004–2015, whereas the female's corresponding trend remained steadily unchanged in Guangzhou. Nevertheless, the incidence rates in 2015 were still higher compared to another nationwide study in China.^31^ The age‐standardized mortality rates for both sexes in Guangzhou showed favorable trends across the entire study period, which were relatively lower than the national mortality data.^31^ Our study elucidated the variation in trends of different cancers. It was illuminating for further investigation of the patterns of incidence and mortality rates and the underlying etiology, natural history, and disparities.

In this study, we observed dramatically ascending trends of incidence rates in thyroid cancer among women and prostate cancer among men, while descending trends in incidence and mortality rates of TBL and nasopharyngeal cancer among men, liver and stomach cancer in both sexes. Meanwhile, incidence and mortality rates of breast cancer for women and colorectal cancer in both sexes presented steady trends but remained relatively high. The identification of joinpoints enabled us to capture the segments of trend changes more precisely. Although the incidence rate of colorectal cancer in males had non‐significant fluctuation with AAPC of 0.4% over the whole study period, a joinpoint was detected in 2012, after which the rate increased rapidly in the following years, insinuating an alarm signal for cancer control. To address this concern, Guangzhou has launched the screening program for colorectal cancer since 2015,^32^ expecting influence on trends in the future.

The corresponding breakdown of effects in age, period, and birth cohort could reveal the influential underlying factors for cancer onset and death trends. Age effect on cancers explicated the significant uprising in incidence and mortality rates with advancing age predominantly among the elderly, suggesting the impact of demographic transition of the aging population.^33^ The patterns were consistent with the national finding and epidemiological studies from two other comparably developed cities, Shanghai and Shenzhen.^34^, ^35^ With an average life expectancy of 81.3 years old, the population in Guangzhou had greater longevity than that nationwide (76.3 years) and worldwide (71.4 years) in 2015. The ongoing evidence suggested a global increase in life expectancy,^36^, ^37^ indicating that the future burden of cancer would continuously increase with aging.

Strong age effects were observed in prostate cancer for males in our study, manifesting that the incidence and mortality rates rose exponentially once aged over 50 years. During the past decade in Guangzhou, prostate cancer incidence and mortality rates have increased substantially, and the trends were compatible with findings in other regions across Asia, such as Hong Kong, Taiwan, Thailand, Japan, and Korea.^38^, ^39^ Prostate cancer was identified with possessing variation with geographical and socio‐economic development, and the commencement of PSA screening and the relevant diagnostic ascertainment were also potentially influential factors.^40^ Guangzhou is a developed city with a prosperous economy, with an increased proportion of the population aged over 65 from 9.6% in 2004 to 11.6% in 2015. The high population density, severe aging population, the development of prostate cancer screening, and the clinical implementation of early diagnosis techniques in Guangzhou's central urban area may contribute to the above‐mentioned elevation.

Cohort effects reflected the diverse risk factors predisposed to different birth cohorts in early life, such as environmental, behavioral, and socio‐economic factors which may manifest a favorable or adverse impact on cancer risk.^16^ Our study found that trends of incidence and mortality rates in the liver and stomach cancer declined among both sexes, demonstrating a continuously decreasing risk in successive birth cohorts. The results were congruous with the trends in China.^41^, ^42^ The underlying etiologies of liver cancer have been recognized, including chronic infection of hepatitis B virus (HBV) and hepatitis C virus (HCV), exposure to aflatoxins, excessive alcohol intake, tobacco smoking, obesity, and diabetes.^43^ The introduction of HBV vaccination for neonates and infants in 1992 played a crucial role in reducing the prevalence of HBV infection from 17.6% to 12.5% in Guangzhou, which was in accordance with the decreased incidence of liver cancer among young generations.^44^, ^45^, ^46^ Measures to improve the environment and optimize the water supply by implementing the Xijiang River Drinking Water Project in Guangzhou contributed to the reduction of risk in liver and stomach cancer.^47^, ^48^

Breast cancer, the most commonly diagnosed malignancy among women in Guangzhou, elucidated the temporal trend of ASIR increased with a non‐significant AAPC of 0.7% for the duration of our study, while noticeable upward trends were reported in the other developed cities, such as Shanghai and Wuhan.^49^, ^50^ The differences may be attributed to cohort effects and period effects. Despite increased risk in the cohort born in the early years, a gradual decline in risk was found in the cohort born from the 1970 s in Guangzhou, leading to a stable tendency of incidence in recent years. However, the incremental rising risk was observed persistently in all birth cohorts among those cities. Compared with the general mammography screening, Guangzhou pioneered establishing a third‐level screening system,^51^ which advanced the sensitivity and specificity of the breast cancer screening program to minimize overdiagnosis.^52^, ^53^

There was a notably steep increase in thyroid cancer incidence rate among women in Guangzhou in the recent 10 years, and APC analysis showed the substantial increase was also driven by both period and birth cohort effects. Previous studies suggested that the rising had been considered to be congruent with the overdiagnosis caused by the extensive use of biopsy techniques, but it was not the sole ascription.^54^ Risk factors of environmental exposure such as iodine deficiency, excess iodine intake, and ionizing radiation should also be scrutinized since the risk was observed among younger generations, which was also reflected in Guangzhou.^55^, ^56^ A plausible reason for the APC model's unidentifiable effects of thyroid cancer mortality was the constantly low mortality rate constantly (less than 1 per 100,000).

Period effects were revealed to have strong impacts on the change in TBL cancer tendency in Guangzhou. Surprising decreasing trends were captured in both age‐standardized incidence and mortality rates of TBL cancer among men during 2004–2015, while the age‐standardized incidence rate was relatively stable among women. Similar patterns were also found in Shanghai and the countries with a high Human Development Index (HDI), such as the United States and Canada.^57^, ^58^ It is reported that smoke‐free ordinances were attributable to reducing new cases of lung cancer.^59^ Guangzhou initially released a “forbidding smoking” notice in public places in 1995, and implemented partial smoke‐free legislation in 2010, contributing to the decrease in smoking prevalence from 46.5% in 2004 to 34.3% in 2011 among men.^60^, ^61^ Disparities of change in incidence trends between sexes may due to the historically negligible smoking rate among females (less than 2%), and exposure to risk factors other than active smoking may play a critical role in the development of lung cancer in females, such as exposure to second‐hand smoking, cooking fume, and air pollutions.^62^, ^63^

In addition, nasopharyngeal carcinoma ranked the fourth leading cancer in Guangzhou, had distinguishable epidemiological features by unique geographic distribution, which was consistent with other research demonstrating the highest incidence and mortality in South Asia, especially the cities and counties in Pearl River Delta.^10^, ^64^ Epstein‐Barr virus (EBV) infection has been widely identified as a vital risk factor in high and low‐incidence regions. Besides, the region‐specific dietary pattern was significantly correlated with the nasopharyngeal carcinoma risk accounting for the large consumption of salted fish and pickled food in southern China, and individuals with a first‐degree family history of nasopharyngeal carcinoma suffered from remarkably higher risk.^64^, ^65^ Although decreasing trends of incidence and mortality rates were observed in Guangzhou, the geographical and familial distribution of nasopharyngeal carcinoma enlightened the necessity of implementing area‐specific primary and secondary prevention for nasopharyngeal carcinoma.

The strengths of this study include that it provided the most updated evaluation of temporal trends for the major cancer incidence and mortality rates in Guangzhou. Not only the changing trends of cancer were assessed, but also the effects of age, cohort, and period were elucidated by the APC model, demonstrating an insight into the analysis of the long‐term registration data and conveying scientific evidence for cancer prevention and ongoing surveillance. Meanwhile, some limitations of our work should be identified. First, our study focused on trend analysis, which was unable to quantify the exposure of risk factors that might contribute to the change in tendencies in cancer. Second, the mortality data in the study period only covered the major districts in Guangzhou instead of the whole city due to the limited availability of cancer registration data in the early years. However, the quality of the data was reassuring. Furthermore, the development and implementation of screening techniques for cancer in recent years might influence the trends of cancer incidence and mortality rates, urging that long‐term effects of trends are of importance to be evaluated continuously in the future.

In summary, the overall age‐standardized incidence and mortality rates showed slightly decreased trends in both sexes in Guangzhou from 2004 to 2015. TBL, colorectal, liver, stomach, nasopharyngeal, prostate, breast, and thyroid cancer are the primary cancers posing a heavy burden of disease for residents in Guangzhou. Trends of incidence and mortality rates were affected by the age, cohort, and period effects, which were varied across cancer sites, reflecting profound impacts of socio‐economic development and lifestyle factors. Therefore, the cancer crisis of human health remains challenging, and it is critically important to establish cancer prevention strategies and public health policies.

## CONFLICTS OF INTEREST

The author declares that there is no conflict of interest.

## AUTHOR CONTRIBUTIONS

AL, HD, XL, YL, BLL, CL, GZL, and YTH participated in study concepts and design; AL, HD, and GZL have contributed to data acquisition; AL, XL, YL, BLL, LC, and YTH have contributed to quality control of data and algorithms; AL, HD, YL, BLL, and LC have contributed to data analysis and interpretation; AL, XL, GZL, and YTH have contributed to statistical analysis; XL, GZL, and YTH are responsible for project administration and supervision; AL, HD, XL, YL, and BLL participated in manuscript preparation and editing; AL, HD, XL, LC, GZL, and YTH have contributed to manuscript review.

## Supporting information

Supplementary Material

## Data Availability

Data retrieved from the Annual Report of Guangzhou Cancer Registry are publicly available on the official website of Guangzhou Municipal Center for Disease Control and Prevention.
