# Supplementary material for: Time trends of major cancers incidence and mortality in Guangzhou, China 2004–2015: A Joinpoint and Age–Period–Cohort Analysis
Source: Cancer Med. 2021 Mar 16;10(8):2865–76. doi: 10.1002/cam4.3744 (PMC8026941; doi:10.1002/cam4.3744)
Supplement: Supplementary file 1 — Supplementary Material [file CAM4-10-2865-s001.docx]

**Supplementary appendix**

**TABLE S1.** Goodness of fit for the age-period-cohort models of major cancers incidence among males in Guangzhou, 2004-2015

**TABLE S2.** Goodness of fit for the age-period-cohort models of major cancers mortality among males in Guangzhou, 2004-2015

**TABLE S3.** Goodness of fit for the age-period-cohort models of major cancers incidence among females in Guangzhou, 2004-2015

**TABLE S4.** Goodness of fit for the age-period-cohort models of major cancers mortality among females in Guangzhou, 2004-2015

**TABLE S1** Goodness of fit for the age-period-cohort models of major cancers incidence among males in Guangzhou, 2004-2015

| **Submodel** |  | **Residual df** | **Residual deviance** | **Comparison** | **Interpretation** | **Change in df** | **Change in deviance** | ***P*-value** |
| --- | --- | --- | --- | --- | --- | --- | --- | --- |
| TBL |  |  |  |  |  |  |  |  |
| 1. Age |  | 161 | 396.43 |  |  |  |  |  |
| 2. Age-drift |  | 160 | 353.65 | 2 vs 1 | Trend (drift) | 1 | 42.79 | <0.001 |
| 3. Age-cohort |  | 155 | 292.64 | 3 vs 2 | Non-linear cohort effect | 5 | 61.01 | <0.001 |
| 4. Age-period |  | 155 | 324.81 | 4 vs 2 | Non-linear period effect | 5 | 28.83 | <0.001 |
| 5. Age-period-cohort |  | 150 | 266.72 | 5 vs 3 | Period effect adjusted for cohort | 5 | 25.92 | <0.001 |
|  |  |  |  | 5 vs 4 | Cohort effect adjusted for period | 5 | 58.09 | <0.001 |
| Liver |  |  |  |  |  |  |  |  |
| 1. Age |  | 161 | 514.47 |  |  |  |  |  |
| 2. Age-drift |  | 160 | 440.97 | 2 vs 1 | Trend (drift) | 1 | 73.51 | <0.001 |
| 3. Age-cohort |  | 155 | 404.64 | 3 vs 2 | Non-linear cohort effect | 5 | 36.33 | <0.001 |
| 4. Age-period |  | 155 | 405.30 | 4 vs 2 | Non-linear period effect | 5 | 35.67 | <0.001 |
| 5. Age-period-cohort |  | 150 | 368.00 | 5 vs 3 | Period effect adjusted for cohort | 5 | 36.63 | <0.001 |
|  |  |  |  | 5 vs 4 | Cohort effect adjusted for period | 5 | 37.29 | <0.001 |
| Colorectal |  |  |  |  |  |  |  |  |
| 1. Age |  | 161 | 241.60 |  |  |  |  |  |
| 2. Age-drift |  | 160 | 241.51 | 2 vs 1 | Trend (drift) | 1 | 0.10 | 0.75 |
| 3. Age-cohort |  | 155 | 220.02 | 3 vs 2 | Non-linear cohort effect | 5 | 21.49 | <0.001 |
| 4. Age-period |  | 155 | 188.09 | 4 vs 2 | Non-linear period effect | 5 | 53.42 | <0.001 |
| 5. Age-period-cohort |  | 150 | 167.83 | 5 vs 3 | Period effect adjusted for cohort | 5 | 52.18 | <0.001 |
|  |  |  |  | 5 vs 4 | Cohort effect adjusted for period | 5 | 20.25 | <0.001 |
| Nasopharyngeal |  |  |  |  |  |  |  |  |
| 1. Age |  | 161 | 418.58 |  |  |  |  |  |
| 2. Age-drift |  | 160 | 266.54 | 2 vs 1 | Trend (drift) | 1 | 152.04 | <0.001 |
| 3. Age-cohort |  | 155 | 263.58 | 3 vs 2 | Non-linear cohort effect | 5 | 2.96 | 0.71 |
| 4. Age-period |  | 155 | 249.69 | 4 vs 2 | Non-linear period effect | 5 | 16.86 | <0.001 |
| 5. Age-period-cohort |  | 150 | 246.21 | 5 vs 3 | Period effect adjusted for cohort | 5 | 17.37 | <0.001 |
|  |  |  |  | 5 vs 4 | Cohort effect adjusted for period | 5 | 3.48 | 0.63 |
| Stomach |  |  |  |  |  |  |  |  |
| 1. Age |  | 161 | 294.85 |  |  |  |  |  |
| 2. Age-drift |  | 160 | 212.37 | 2 vs 1 | Trend (drift) | 1 | 82.48 | <0.001 |
| 3. Age-cohort |  | 155 | 191.38 | 3 vs 2 | Non-linear cohort effect | 5 | 20.99 | <0.001 |
| 4. Age-period |  | 155 | 204.82 | 4 vs 2 | Non-linear period effect | 5 | 7.55 | 0.18 |
| 5. Age-period-cohort |  | 150 | 183.55 | 5 vs 3 | Period effect adjusted for cohort | 5 | 7.83 | 0.17 |
|  |  |  |  | 5 vs 4 | Cohort effect adjusted for period | 5 | 21.27 | <0.001 |
| Prostate |  |  |  |  |  |  |  |  |
| 1. Age |  | 162 | 248.16 |  |  |  |  |  |
| 2. Age-drift |  | 161 | 175.67 | 2 vs 1 | Trend (drift) | 1 | 72.49 | <0.001 |
| 3. Age-cohort |  | 156 | 165.81 | 3 vs 2 | Non-linear cohort effect | 5 | 9.86 | 0.08 |
| 4. Age-period |  | 156 | 169.86 | 4 vs 2 | Non-linear period effect | 5 | 5.81 | 0.33 |
| 5. Age-period-cohort |  | 151 | 161.93 | 5 vs 3 | Period effect adjusted for cohort | 5 | 3.88 | 0.57 |
|  |  |  |  | 5 vs 4 | Cohort effect adjusted for period | 5 | 7.94 | 0.16 |

**TABLE S2** Goodness of fit for the age-period-cohort models of major cancers mortality among males in Guangzhou, 2004-2015

| **Submodel** |  | **Residual df** | **Residual deviance** | **Comparison** | **Interpretation** | **Change in df** | **Change in deviance** | ***P*-value** |
| --- | --- | --- | --- | --- | --- | --- | --- | --- |
| TBL |  |  |  |  |  |  |  |  |
| 1. Age |  | 162 | 643.29 |  |  |  |  |  |
| 2. Age-drift |  | 161 | 460.66 | 2 vs 1 | Trend (drift) | 1 | 182.63 | <0.001 |
| 3. Age-cohort |  | 156 | 391.80 | 3 vs 2 | Non-linear cohort effect | 5 | 68.86 | <0.001 |
| 4. Age-period |  | 156 | 337.25 | 4 vs 2 | Non-linear period effect | 5 | 123.41 | <0.001 |
| 5. Age-period-cohort |  | 151 | 260.86 | 5 vs 3 | Period effect adjusted for cohort | 5 | 130.94 | <0.001 |
|  |  |  |  | 5 vs 4 | Cohort effect adjusted for period | 5 | 76.39 | <0.001 |
| Liver |  |  |  |  |  |  |  |  |
| 1. Age |  | 161 | 452.41 |  |  |  |  |  |
| 2. Age-drift |  | 160 | 310.35 | 2 vs 1 | Trend (drift) | 1 | 142.06 | <0.001 |
| 3. Age-cohort |  | 155 | 282.56 | 3 vs 2 | Non-linear cohort effect | 5 | 27.79 | <0.001 |
| 4. Age-period |  | 155 | 267.79 | 4 vs 2 | Non-linear period effect | 5 | 42.56 | <0.001 |
| 5. Age-period-cohort |  | 150 | 237.24 | 5 vs 3 | Period effect adjusted for cohort | 5 | 45.32 | <0.001 |
|  |  |  |  | 5 vs 4 | Cohort effect adjusted for period | 5 | 30.55 | <0.001 |
| Colorectal |  |  |  |  |  |  |  |  |
| 1. Age |  | 161 | 241.14 |  |  |  |  |  |
| 2. Age-drift |  | 160 | 241.11 | 2 vs 1 | Trend (drift) | 1 | 0.03 | 0.86 |
| 3. Age-cohort |  | 155 | 221.27 | 3 vs 2 | Non-linear cohort effect | 5 | 19.84 | <0.001 |
| 4. Age-period |  | 155 | 199.48 | 4 vs 2 | Non-linear period effect | 5 | 41.62 | <0.001 |
| 5. Age-period-cohort |  | 150 | 178.14 | 5 vs 3 | Period effect adjusted for cohort | 5 | 43.14 | <0.001 |
|  |  |  |  | 5 vs 4 | Cohort effect adjusted for period | 5 | 21.35 | <0.001 |
| Nasopharyngeal |  |  |  |  |  |  |  |  |
| 1. Age |  | 161 | 228.88 |  |  |  |  |  |
| 2. Age-drift |  | 160 | 171.39 | 2 vs 1 | Trend (drift) | 1 | 57.49 | <0.001 |
| 3. Age-cohort |  | 155 | 169.29 | 3 vs 2 | Non-linear cohort effect | 5 | 2.10 | 0.83 |
| 4. Age-period |  | 155 | 153.45 | 4 vs 2 | Non-linear period effect | 5 | 17.93 | <0.001 |
| 5. Age-period-cohort |  | 150 | 151.33 | 5 vs 3 | Period effect adjusted for cohort | 5 | 17.96 | <0.001 |
|  |  |  |  | 5 vs 4 | Cohort effect adjusted for period | 5 | 2.12 | 0.83 |
| Stomach |  |  |  |  |  |  |  |  |
| 1. Age |  | 161 | 241.75 |  |  |  |  |  |
| 2. Age-drift |  | 160 | 166.34 | 2 vs 1 | Trend (drift) | 1 | 75.41 | <0.001 |
| 3. Age-cohort |  | 155 | 157.93 | 3 vs 2 | Non-linear cohort effect | 5 | 8.42 | 0.13 |
| 4. Age-period |  | 155 | 152.31 | 4 vs 2 | Non-linear period effect | 5 | 14.03 | 0.02 |
| 5. Age-period-cohort |  | 150 | 143.48 | 5 vs 3 | Period effect adjusted for cohort | 5 | 14.44 | 0.01 |
|  |  |  |  | 5 vs 4 | Cohort effect adjusted for period | 5 | 8.83 | 0.12 |
| Prostate |  |  |  |  |  |  |  |  |
| 1. Age |  | 163 | 146.27 |  |  |  |  |  |
| 2. Age-drift |  | 162 | 131.17 | 2 vs 1 | Trend (drift) | 1 | 15.10 | <0.001 |
| 3. Age-cohort |  | 157 | 128.01 | 3 vs 2 | Non-linear cohort effect | 5 | 3.16 | 0.68 |
| 4. Age-period |  | 157 | 110.73 | 4 vs 2 | Non-linear period effect | 5 | 20.44 | <0.001 |
| 5. Age-period-cohort |  | 152 | 107.97 | 5 vs 3 | Period effect adjusted for cohort | 5 | 20.04 | <0.001 |
|  |  |  |  | 5 vs 4 | Cohort effect adjusted for period | 5 | 2.76 | 0.74 |

**TABLE S3** Goodness of fit for the age-period-cohort models of major cancers incidence among females in Guangzhou, 2004-2015

| **Submodel** |  | **Residual df** | **Residual deviance** | **Comparison** | **Interpretation** | **Change in df** | **Change in deviance** | ***P*-value** |
| --- | --- | --- | --- | --- | --- | --- | --- | --- |
| Breast |  |  |  |  |  |  |  |  |
| 1. Age |  | 161 | 616.39 |  |  |  |  |  |
| 2. Age-drift |  | 160 | 602.85 | 2 vs 1 | Trend (drift) | 1 | 13.54 | <0.001 |
| 3. Age-cohort |  | 155 | 504.49 | 3 vs 2 | Non-linear cohort effect | 5 | 98.36 | <0.001 |
| 4. Age-period |  | 155 | 585.61 | 4 vs 2 | Non-linear period effect | 5 | 17.24 | <0.001 |
| 5. Age-period-cohort |  | 150 | 485.85 | 5 vs 3 | Period effect adjusted for cohort | 5 | 18.64 | <0.001 |
|  |  |  |  | 5 vs 4 | Cohort effect adjusted for period | 5 | 99.76 | <0.001 |
| TBL |  |  |  |  |  |  |  |  |
| 1. Age |  | 161 | 578.29 |  |  |  |  |  |
| 2. Age-drift |  | 160 | 556.57 | 2 vs 1 | Trend (drift) | 1 | 21.71 | <0.001 |
| 3. Age-cohort |  | 155 | 506.53 | 3 vs 2 | Non-linear cohort effect | 5 | 50.05 | <0.001 |
| 4. Age-period |  | 155 | 492.24 | 4 vs 2 | Non-linear period effect | 5 | 64.34 | <0.001 |
| 5. Age-period-cohort |  | 150 | 436.65 | 5 vs 3 | Period effect adjusted for cohort | 5 | 69.87 | <0.001 |
|  |  |  |  | 5 vs 4 | Cohort effect adjusted for period | 5 | 55.59 | <0.001 |
| Colorectal |  |  |  |  |  |  |  |  |
| 1. Age |  | 161 | 453.40 |  |  |  |  |  |
| 2. Age-drift |  | 160 | 451.85 | 2 vs 1 | Trend (drift) | 1 | 1.55 | 0.21 |
| 3. Age-cohort |  | 155 | 382.73 | 3 vs 2 | Non-linear cohort effect | 5 | 69.13 | <0.001 |
| 4. Age-period |  | 155 | 413.05 | 4 vs 2 | Non-linear period effect | 5 | 38.81 | <0.001 |
| 5. Age-period-cohort |  | 150 | 341.33 | 5 vs 3 | Period effect adjusted for cohort | 5 | 41.40 | <0.001 |
|  |  |  |  | 5 vs 4 | Cohort effect adjusted for period | 5 | 71.72 | <0.001 |
| Liver |  |  |  |  |  |  |  |  |
| 1. Age |  | 161 | 333.69 |  |  |  |  |  |
| 2. Age-drift |  | 160 | 319.23 | 2 vs 1 | Trend (drift) | 1 | 14.45 | <0.001 |
| 3. Age-cohort |  | 155 | 269.42 | 3 vs 2 | Non-linear cohort effect | 5 | 49.81 | <0.001 |
| 4. Age-period |  | 155 | 281.28 | 4 vs 2 | Non-linear period effect | 5 | 37.96 | <0.001 |
| 5. Age-period-cohort |  | 150 | 229.91 | 5 vs 3 | Period effect adjusted for cohort | 5 | 39.51 | <0.001 |
|  |  |  |  | 5 vs 4 | Cohort effect adjusted for period | 5 | 51.37 | <0.001 |
| Stomach |  |  |  |  |  |  |  |  |
| 1. Age |  | 161 | 347.89 |  |  |  |  |  |
| 2. Age-drift |  | 160 | 282.16 | 2 vs 1 | Trend (drift) | 1 | 65.73 | <0.001 |
| 3. Age-cohort |  | 155 | 258.09 | 3 vs 2 | Non-linear cohort effect | 5 | 24.07 | <0.001 |
| 4. Age-period |  | 155 | 243.14 | 4 vs 2 | Non-linear period effect | 5 | 39.02 | <0.001 |
| 5. Age-period-cohort |  | 150 | 218.73 | 5 vs 3 | Period effect adjusted for cohort | 5 | 39.36 | <0.001 |
|  |  |  |  | 5 vs 4 | Cohort effect adjusted for period | 5 | 24.40 | <0.001 |
| Thyroid |  |  |  |  |  |  |  |  |
| 1. Age |  | 161 | 1518.85 |  |  |  |  |  |
| 2. Age-drift |  | 160 | 283.64 | 2 vs 1 | Trend (drift) | 1 | 1235.21 | <0.001 |
| 3. Age-cohort |  | 155 | 219.13 | 3 vs 2 | Non-linear cohort effect | 5 | 64.51 | <0.001 |
| 4. Age-period |  | 155 | 254.39 | 4 vs 2 | Non-linear period effect | 5 | 29.25 | <0.001 |
| 5. Age-period-cohort |  | 150 | 193.61 | 5 vs 3 | Period effect adjusted for cohort | 5 | 25.52 | <0.001 |
|  |  |  |  | 5 vs 4 | Cohort effect adjusted for period | 5 | 60.78 | <0.001 |

**TABLE S4** Goodness of fit for the age-period-cohort models of major cancers mortality among females in Guangzhou, 2004-2015

| **Submodel** |  | **Residual df** | **Residual deviance** | **Comparison** | **Interpretation** | **Change in df** | **Change in deviance** | ***P*-value** |
| --- | --- | --- | --- | --- | --- | --- | --- | --- |
| Breast |  |  |  |  |  |  |  |  |
| 1. Age |  | 161 | 183.64 |  |  |  |  |  |
| 2. Age-drift |  | 160 | 182.21 | 2 vs 1 | Trend (drift) | 1 | 1.42 | 0.23 |
| 3. Age-cohort |  | 155 | 178.88 | 3 vs 2 | Non-linear cohort effect | 5 | 3.33 | 0.65 |
| 4. Age-period |  | 155 | 160.07 | 4 vs 2 | Non-linear period effect | 5 | 22.14 | <0.001 |
| 5. Age-period-cohort |  | 150 | 156.69 | 5 vs 3 | Period effect adjusted for cohort | 5 | 22.19 | <0.001 |
|  |  |  |  | 5 vs 4 | Cohort effect adjusted for period | 5 | 3.38 | 0.64 |
| TBL |  |  |  |  |  |  |  |  |
| 1. Age |  | 161 | 432.19 |  |  |  |  |  |
| 2. Age-drift |  | 160 | 345.11 | 2 vs 1 | Trend (drift) | 1 | 87.07 | <0.001 |
| 3. Age-cohort |  | 155 | 314.15 | 3 vs 2 | Non-linear cohort effect | 5 | 30.97 | <0.001 |
| 4. Age-period |  | 155 | 291.19 | 4 vs 2 | Non-linear period effect | 5 | 53.92 | <0.001 |
| 5. Age-period-cohort |  | 150 | 259.93 | 5 vs 3 | Period effect adjusted for cohort | 5 | 54.21 | <0.001 |
|  |  |  |  | 5 vs 4 | Cohort effect adjusted for period | 5 | 31.26 | <0.001 |
| Colorectal |  |  |  |  |  |  |  |  |
| 1. Age |  | 161 | 216.99 |  |  |  |  |  |
| 2. Age-drift |  | 160 | 215.76 | 2 vs 1 | Trend (drift) | 1 | 1.23 | 0.27 |
| 3. Age-cohort |  | 155 | 211.63 | 3 vs 2 | Non-linear cohort effect | 5 | 4.13 | 0.53 |
| 4. Age-period |  | 155 | 175.48 | 4 vs 2 | Non-linear period effect | 5 | 40.28 | <0.001 |
| 5. Age-period-cohort |  | 150 | 171.51 | 5 vs 3 | Period effect adjusted for cohort | 5 | 40.12 | <0.001 |
|  |  |  |  | 5 vs 4 | Cohort effect adjusted for period | 5 | 3.98 | 0.55 |
| Liver |  |  |  |  |  |  |  |  |
| 1. Age |  | 161 | 264.63 |  |  |  |  |  |
| 2. Age-drift |  | 160 | 195.80 | 2 vs 1 | Trend (drift) | 1 | 68.83 | <0.001 |
| 3. Age-cohort |  | 155 | 189.75 | 3 vs 2 | Non-linear cohort effect | 5 | 6.05 | 0.30 |
| 4. Age-period |  | 155 | 164.24 | 4 vs 2 | Non-linear period effect | 5 | 31.56 | <0.001 |
| 5. Age-period-cohort |  | 150 | 158.25 | 5 vs 3 | Period effect adjusted for cohort | 5 | 31.50 | <0.001 |
|  |  |  |  | 5 vs 4 | Cohort effect adjusted for period | 5 | 5.99 | 0.31 |
| Stomach |  |  |  |  |  |  |  |  |
| 1. Age |  | 161 | 199.14 |  |  |  |  |  |
| 2. Age-drift |  | 160 | 172.52 | 2 vs 1 | Trend (drift) | 1 | 26.62 | <0.001 |
| 3. Age-cohort |  | 155 | 159.17 | 3 vs 2 | Non-linear cohort effect | 5 | 13.34 | 0.02 |
| 4. Age-period |  | 155 | 158.58 | 4 vs 2 | Non-linear period effect | 5 | 13.94 | 0.02 |
| 5. Age-period-cohort |  | 150 | 144.62 | 5 vs 3 | Period effect adjusted for cohort | 5 | 14.55 | 0.01 |
|  |  |  |  | 5 vs 4 | Cohort effect adjusted for period | 5 | 13.96 | 0.02 |
| Thyroid |  |  |  |  |  |  |  |  |
| 1. Age |  | 161 | 122.46 |  |  |  |  |  |
| 2. Age-drift |  | 160 | 121.27 | 2 vs 1 | Trend (drift) | 1 | 1.19 | 0.27 |
| 3. Age-cohort |  | 155 | 115.60 | 3 vs 2 | Non-linear cohort effect | 5 | 5.67 | 0.34 |
| 4. Age-period |  | 155 | 116.78 | 4 vs 2 | Non-linear period effect | 5 | 4.49 | 0.48 |
| 5. Age-period-cohort |  | 150 | 110.70 | 5 vs 3 | Period effect adjusted for cohort | 5 | 4.89 | 0.43 |
|  |  |  |  | 5 vs 4 | Cohort effect adjusted for period | 5 | 6.08 | 0.30 |
